# A Fatal Case of Primary Orbital Ewing’s Sarcoma with Intracranial Extensions in a Six-year-old Girl

**DOI:** 10.7759/cureus.6248

**Published:** 2019-11-27

**Authors:** Natasha Raza, Zia Iqbal, Mahwish Nasim, Saad Nasir, Alina Sehar

**Affiliations:** 1 Pediatrics, Civil Hospital Karachi, Dow University of Health Sciences, Karachi, PAK; 2 Pediatrics, Dow University of Health Sciences, Karachi, PAK; 3 Internal Medicine, United Medical and Dental College/Creek General Hospital, Karachi, PAK; 4 Internal Medicine, United Medical and Dental College, Karachi, PAK

**Keywords:** orbital ewing sarcoma, ewing sarcoma, bone tumor

## Abstract

Ewing’s sarcoma (ES) is the second most common skeletal tumor seen in children and adolescents which typically involves the long bones and the axial skeleton. ES involving the orbits is extremely rare and can lead to serious consequences. Patients usually present with fever, localized bone pain which increases during the night time, and a visible mass, often with a preceding history of trauma. The diagnosis is confirmed with immunohistochemistry. Patient management involves a multidisciplinary approach with complete focal surgical excision of the tumor along with multiple chemotherapeutic agents and radiation therapy.

## Introduction

Ewing’s sarcoma (ES) is the second^ ^most prevailing malignant skeletal tumor in children and teenagers, which may also originate in soft tissues [1]. An extremely aggressive tumor, ES has a likelihood of mortality in 20%-30% of patients who have focal involvement and around 70% in patients with metastasis [2]. Out of 253 cases of pediatric malignant solid tumors in Pakistan, 4.5% of the patients were identified with ES [3]. The most frequent primary site of ES involves the lower extremities (45%), pelvis (20%), upper extremities (13%), axial skeleton, ribs (13%), and face (2%) [4]. A primary ES of the head and neck area is uncommon and if present, it most frequently occurs in the mandible or maxilla [5]. Primary ES of the orbits is extremely rare and is often associated with unilateral proptosis; ipsilateral to the tumor site [6]. We hereby present a unique case of primary orbital ES presenting with unilateral proptosis of the left eye due to intracranial extensions in a six-year-old girl.

## Case presentation

A six-year-old girl was brought by her mother to the pediatric outpatient department of a tertiary care hospital in Karachi with a 15-day history of a persistent dull headache. The headache was more prominent on the left side of her face and was associated with nausea. Her mother also reported that since the past three days, she was unable to open her left eye. She had a swelling on the left side of the face, extending behind the ear up to the jawline. 

The general physical examination was unremarkable. She was alert and oriented to time and place. Her vitals were taken which showed a heart rate of 96/min, respiratory rate of 30/min, afebrile and blood pressure of 100/70 mmHg. Her random blood sugar was 97 mg/dl. Pertinent examination finding included two palpable jugulodigastric nodes and the largest was more than 1 cm in size, soft in consistency, and neither was matted. Systemic examination revealed ptosis of the left eye with mild proptosis along with the presence of external and internal ophthalmoplegia. Her pupils were unequal, dilated, sluggishly reactive to light. The right pupil was reactive to light with intact direct and consensual reflexes, as compared to the absence of both reflexes in the left pupil. Systemic examination otherwise was unremarkable. Initially, the patient was managed with continuous observation for signs of raised intracranial pressure (ICP) and blood pressure was monitored periodically.

Her laboratory investigations were as follows: hemoglobin (Hb) 12.6 mg/dl (normal range: 12.0-15.5 mg/dl), total leukocyte count (TLC): 11200/mm^3^ (normal range: 3600-11,200/mm^3^), erythrocyte sedimentation rate (ESR): 5 mm 1st hour (normal: <20 mm/hr), blood urea nitrogen (BUN): 7 mg/dl (age-appropriate normal range: 7-10 mg/dl), creatinine: 0.2 mg/dl (age-appropriate normal range: 0.3-0.5 mg/dl), sodium: 135 mEq/L (normal range: 135-145 mEq/L), potassium: 3.9 mEq/L (normal range: 3.5-5.0 mEq/L), chloride: 98 mEq/L (normal range: 96-106 mEq/L), calcium: 9.8 mg/dl (normal range: 8.5-10.5 mg/dl), magnesium: 2 mEq/L (normal range: 1.5-2.3 mEq/L), uric acid: 2 mg/dl (normal range: 2.0-5.5 mg/dl), lactate dehydrogenase (LDH): 110 IU/L (normal range: 60-170 IU/L). During the course of her hospital stay, intravenous (IV) ceftriaxone, IV vancomycin and IV metronidazole were administered on suspicion of brain abscess.

**Figure 1:**
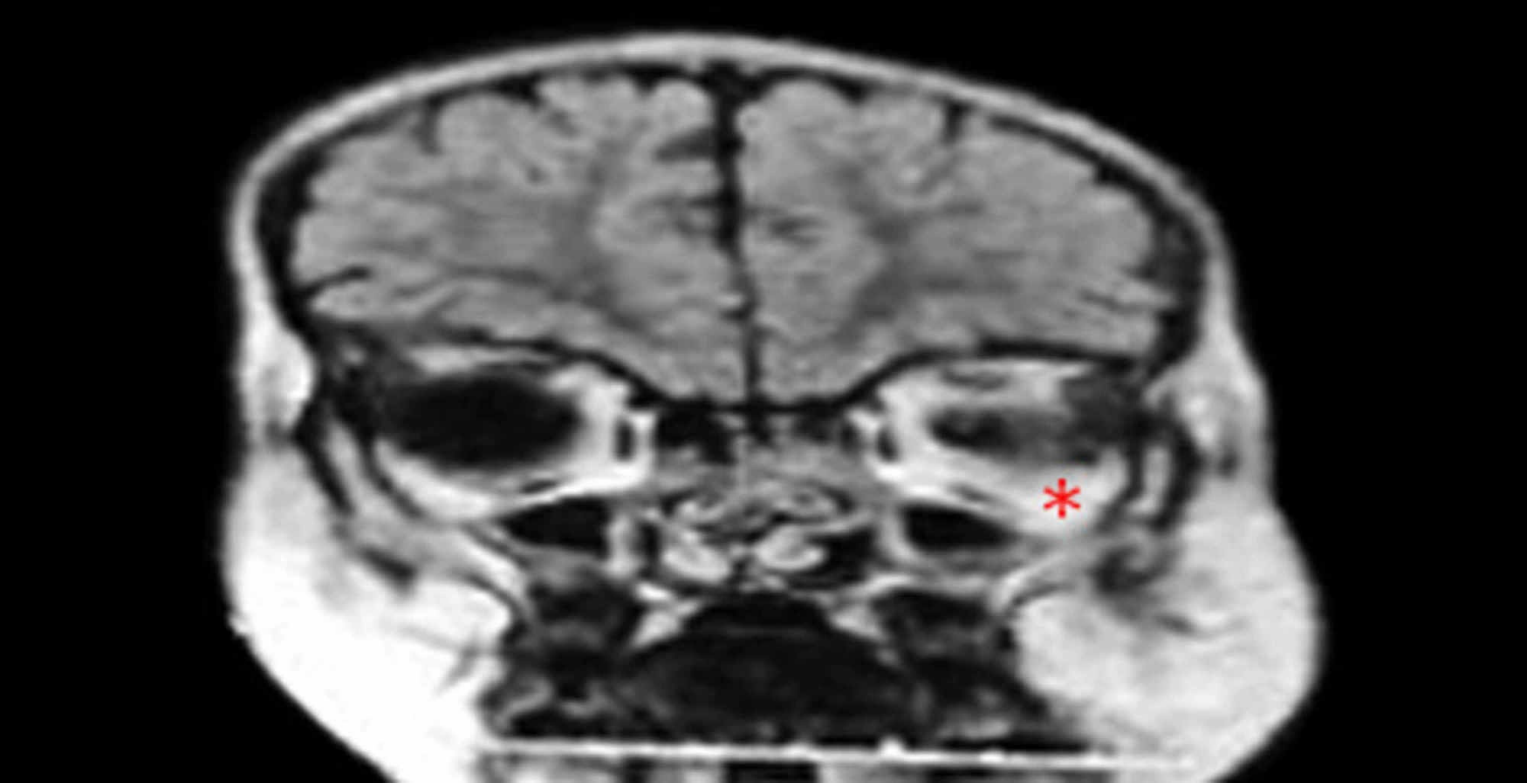
Magnetic resonance imaging (MRI) coronal view showing a well-defined abnormal single intensity lesion in the left temporal and left masticator space involving the medial pterigoid, lateral pterigyoid, masseter, and temporalis muscles; it also involves the left maxilla, infratemporal area, left parapharyngeal space, and left oropharynx area *= Tumor

A consultation for ophthalmology findings was taken, which showed normal visual acuity of 6/6 in both eyes. Her right eye appeared normal and the left eye had complete ptosis with a frozen eyeball, dilated pupil and mild proptosis. Vitreous media was clear and fundus was normal. The patient was suspected to have “orbital apex syndrome”, which is defined as the simultaneous dysfunction of the optic nerve and the cranial nerves (manifesting with vision loss, ptosis, and a complete internal and external ophthalmoplegia) as a result of a process occurring in the region of the optic canal and the superior orbital fissure. On neurological consultation, plain computed tomography (CT) scan of the brain was normal, while CT scan with contrast of the brain showed no space-occupying lesion. The patient was advised to undergo an urgent magnetic resonance imaging (MRI), which revealed a well-defined abnormal single intensity lesion in left masticator space involving the medial pterygoid, lateral pterygoid, masseter and temporalis muscles causing pressure remodeling of the pterygoid plate, and returning hypointense signals on T1-weighted images (T1WI), heterogeneously hyper-intense signals on T2-weighted images (T2WI), and fluid-attenuated inversion recovery (FLAIR) sequences without restriction on diffusion-weighted magnetic resonance imaging (DWI-MRI) and showed heterogeneous post-contrast enhancement with foci necrosis, which measured 5.9 x 4.2 x 4.1 cm (Figures 1). Superiorly, it was extending into infratemporal fossa causing widening of pterygomaxillary fissures and also into extradural space of left middle cranial fossa through foramen ovale involving adjacent dura matter. It was also involving the left cavernous sinus encasing and displacing the internal carotid artery and blocking the left optic canal. Laterally, it was causing pressure remodeling of the left mandibular ramus. Inferiorly, it was extending up to the mandibular angle. There was no midline shift and ventricles were of the normal size. The findings were suggestive of an aggressive lesion in left masticator space with intracranial and cavernous sinus extension. The differential diagnosis that was considered included schwannoma, neurofibroma, and rhabdomyosarcoma; histopathology was recommended for confirmation.

On histopathology, the lower pole was undifferentiated and densely cellular with light and dark cell appearance with sheets of small, round, uniform cells with scant clear cytoplasm. These cells frequently contained amphophilic glycogen vacuoles that were divided into irregular lobules by fibrous strands. There was also the presence of large pleomorphic cells in an organoid pattern, with large areas of perivascular tumor necrosis. These findings were suggestive of ES. Immunohistochemistry was subsequently done and showed positive results for cluster of differentiation 99 (CD99) and friend leukemia integration 1 transcription factor (FLI-1) tumor markers, thus confirming the diagnosis of ES. Due to widespread metastasis (stage IV) of the tumor, the patient was kept on palliative care and the parents were counselled about the poor prognosis. The parents decided to leave against medical advice (AMA). Unfortunately, the patient died 10 days later.

## Discussion

ES, described in 1921 by James Ewing, is a member of a family of small blue round cell tumors that remain undifferentiated. It most frequently involves bone, but may also sometimes appear in soft tissues, with a strong likelihood of early metastasis at presentation. A rough incidence of ES was approximated to be 1.5 cases /1,000,000 in people of European descent, 0.8 cases/ 1,000,000 in Asians and 0.2 cases/ 1,000,000 in people of African descent [2]. Ahmad et al. determined the overall frequency of ES in Pakistan to be 1.2% of all tumors, out of which 43.7% and 52.9% had bone and soft tissue involvement, respectively [7]. Findings that confirm the diagnosis of ES, using immunohistochemistry, show positive results for CD99 and FLI-1 tumor markers [8]. 

To the best of our knowledge, there have only been two similar cases of children (13-year-old girl [9], 16-year-old girl [10]) presenting with a primary orbital ES, in Pakistan; however, our case was different because it was rapidly progressive and the patient died within days after the diagnosis. There have been other case reports of orbital ES with intracranial extension from other regions including India (13-year-old girl) [11], Italy (16-year-old boy) [5], and Canada (6-year-old boy) [6].

Multiple studies regarding the use of numerous treatment modalities have shown the effectiveness of this approach with clear evidence of better outcomes in patients with orbital ES [9,12]. In a cohort study done in the United States, 42 patients aged less than 10 years were identified with ES [13]. The study showed a preponderance of ES in males (62%), as compared to females (38%). The most frequently involved primary site were the extremities (41%), while the incidence of head and neck primary ES was determined to be 19%. The overall survival rate was significantly high and determined to be 82% (95% confidence interval: 71%-95%), with only metastasis at the time of diagnosis serving as a reliable prognostic factor [13]. In another cohort study, comparison for five-year overall survival was drawn between adult and pediatric populations with ES and was determined to be 43% for adults and 66% for children (P<0.001) [14].

## Conclusions

A primary orbital ES with intracranial extensions manifesting as unilateral proptosis is extremely rare in children and has a good prognosis if prompt treatment using a multi-disciplinary approach is initiated. Although rare, ES can also be rapidly progressive and fatal. Physicians should keep a low threshold for early recognition of ES in the pediatric population presenting with fever, localized bone pain, proptosis, or orbital swelling.
